# Occurrence of colorectal aberrant crypt foci depending on age and dietary patterns of patients

**DOI:** 10.1186/s12885-018-4100-8

**Published:** 2018-02-21

**Authors:** Marek Kowalczyk, Marcin Orłowski, Piotr Siermontowski, Dariusz Mucha, Krzysztof Zinkiewicz, Waldemar Kurpiewski, Ewa Zieliński, Iwona Kowalczyk, Agnieszka Pedrycz

**Affiliations:** 1 Department of Oncologic and General Surgery, University Hospital in Olsztyn, Olsztyn, Poland; 2 Centre for Diagnosis and Treatment of Gastrointestinal Diseases, Gdańsk, Poland; 3 Naval Academy, Underwater Works Technology Department, Gdynia, Poland; 4 Department of Physical Education and Sport, Academy of Physical Education in Cracow, Cracow, Poland; 5 2nd Department of General, Gastroenterologic and Gastrointestinal Oncologic Surgery, Medical University of Lublin, University Hospital No.1, Lublin, Poland; 6 Department of Emergency Medicine and Disaster Collegium Medicum in Bydgoszcz, Nicolaus Copernicus University in Toruń, Bydgoszcz, Poland; 7 Unit for Laboratory Diagnostics, University Hospital in Olsztyn, Olsztyn, Poland; 8 Department of Histology and Embryology with Unit of Experimental Cytology, Medical University of Lublin, Lublin, Poland

**Keywords:** Aberrant crypt foci, Adenoma, Large intestine, Dietary patterns

## Abstract

**Background:**

Aberrant crypt foci (ACF) are commonly considered the early pre-cancerous lesions that can progress to colorectal cancer (CRC). The available literature data reveal that age, dietary factors and lifestyle can affect the development of several dozen percentages of malignant tumours, including CRC. In the present study, an attempt was made to assess the incidence and growth dynamics of ACF and to determine whether the type of diet affected the development and number of AFC.

**Methods:**

Colonoscopy combined with rectal mucosa staining with 0.25% methylene blue was performed in 131 patients. On the day of examination, each patient completed a questionnaire regarding epidemiological data. According to their numbers, colorectal ACF were divided into three groups. The findings were analysed statistically. The Student’s t test and the U test were applied in order to determine the significance of differences of means and frequency of events in both groups. Statistica 7.1 and Excel 2010 were used.

**Results:**

The single ACF occur in the youngest individuals (ACF < 5). Since the age of 38 years, the number of ACF gradually increases to show a decreasing tendency since the age of 60 years. The number of 5 < ACF < 10 occurs slightly later, since the age of 50 years, and dynamically increases reaching the maximum at the age of 62 years, subsequently the increase is proportional. ACF > 10 occur at a more advanced age (55 years) and their number gradually increases with age. The maximum number is observed at the age of 77 years.

In individuals not using high-fibre diets and with high intake of red meat, the probability of higher numbers of ACF increases. The probability of higher numbers of ACF (5 < ACF10) was observed in patients with colon diverticula. In patients with higher BMI, the number of ACF is higher.

**Conclusion:**

Age significantly affects the number of colorectal ACF. The types of foods consumed can considerably increase the risk of colorectal ACF, which is particularly visible in individuals who do not regularly use high-fibre diets, those obese and with colon diverticula.

## Background

Aberrant crypt foci (ACF) were first described by Bird in mice exposed to a mutagenic agent – azoxymethane in 1987 [1]. Colorectal ACF are commonly considered the early pre-cancerous lesions that can progress to colorectal cancer. According to the literature data, large geographical differences in the incidence of colorectal cancer result from environmental and dietary factors or lifestyle [2–9]. The literature findings demonstrate that age, dietary factors and lifestyle are likely to affect the development of even several dozen percentages of malignant cancers [4, 5]. Diets play a key role. It is generally believed that diets rich in animal fat and red meat increase the risk of gastrointestinal cancers while diets rich in fresh fruit and vegetables distinctly reduce this risk [4–6].

The factors increasing the risk of colorectal cancer can be divided into four categories:

1. Epidemiological factors: age, race, body weight and physical activity, geographical and family-related factors
2. Hereditary factors (intestinal):
  genetically conditioned syndromes leading to the development of cancer
  history of adenomatous polyps or colorectal cancer
  non-specific inflammatory intestinal diseases
3. Dietary factors:
  high-fat, high-protein and low-fibre diets
  protective effects of vitamins and antioxidants
4. Mixed factors:
  ureterosigmoidostomy
  post-cholecystectomy and post-abdominal radiation therapy conditions
  acromegaly.

A risk factor is any possible condition significantly increasing or reducing the risk of malignant tumour (which is confirmed statistically); hence, there are numerous elements that influence the risk of cancer. Countless products of bacterial fermentation of food residues occur in the colon; therefore, it is essential that the colon is intact and that the colonic wall is not exposed to potentially harmful factors. The epithelial barrier of the colon is resistant to the effects of damaging factors. It separates the colonic lumen from the lamina propria and the vascular system. The specialised epithelial cells are covered by a double layer rich in phospholipids; they adhere closely and contain various connections involved in the transport of ions and water. Moreover, E-cadherin- and desmosome-dependent connections strengthen this barrier. The compounds exerting protective effects are called anticarcinogens or antimutagens [5]. The dietary components can act at various stages of carcinogenesis. The dietary antimutagens can be classified into two groups: bioantimutagens, which affect DNA and desmutagens that act indirectly on the genetic material. Bioantimutagens are the naturally occurring substances, which reduce the number of mutations by participating in DNA repair processes. Desmutagens involve all the factors, whose effects are not connected with DNA repair or replication. They induce various enzymes, remove mutagens and block the activation of mutagens.

In their controlled clinical trial, Evans et al. have demonstrated protective effects of fruit and vegetables containing galactose-rich fibres on the development of colorectal cancer as the galactose residues can bind mitogenic lectins (plant–derived) and thus prevent the development of colorectal cancer. Furthermore, a significant role is attributed to dietary fibre [10]. The plant fibre increases the production of volatile fatty acids, including derivatives of butyric acid, which protect the mucous membrane against dysplastic transformation. Thanks to the presence of volatile fatty acids, pH of the faeces is low and the degradation of sterols reduced. Another important antineoplastic factor is accelerated desquamation of the mucosal cells mediated by pectin-sterol complexes [4]. The individuals on diets with excessive amounts of fats and proteins yet deficient in fibre are characterised by increased secretion of biliary bile acids, whose metabolites produced by anaerobic bacteria, especially *Clostridium, Streptococcus, Bacteroides, Bifidobacterium*, change the intestinal microflora and promote carcinogenesis in the colon. The secondary biliary acids produced, e.g. taurodeoxycholic and litocholic acids, exert pro-carcinogenic effects [11]. Moreover, some other intestinal bacteria and yeast are of interest as well.

The role of a diet in the development of colorectal cancer has not been fully elucidated. The epidemiological data indicate that consumption of red meat, which components can contribute to the development of cancer, is of importance. The thermal preparation of meat (boiling, grilling) releases heterocyclic amines that can damage DNA of colonocytes [4, 12–14]. Furthermore, animal fats often accompanying the red meat consumed are responsible for intra-intestinal concentration of biliary and fatty acids that can damage the cell membranes [15]. Both of these factors acting simultaneously increase the iron content in the colonic lumen [16, 17]. Excessive amounts of unabsorbed iron can induce the undesirable Fenton phenomenon [18, 19]. High intake of vegetables and fruit rich in vitamins, such as β-carotene and vitamin C, which are natural antioxidants, can prevent free radical-dependent damage to colonocytes [5].

Many of the studies mentioned above implicate that the composition of diets significantly affects the development of colorectal cancer and its potential precursors, i.e. ACF.

## Aim

In the present study, an attempt was made to evaluate the incidence of ACF and dynamics of their growth in individual age groups and to determine whether the type of diet affected the formation and number of ACF.

## Methods

The project was approved by the Bioethics Committee at the Faculty of Medical Sciences of the University of Warmia and Mazury in Olsztyn - Resolution No. 11/2010 of 29 April 2010. Colonoscopy combined with rectal mucosa staining with 0.25% methylene blue was performed in 131 patients. Each of the study participants gave informed consent to participate in the study. Three bioptates were collected from the foci defined macroscopically as ACF; in cases where there were fewer foci, the number of collected foci was respectively lower. On the colonoscopy day, patients completed the questionnaire regarding epidemiological data used for analysis of factors affecting the occurrence of ACF in the study group. The number of ACF in the colon was divided into three groups:

- ACF < 5
- 5 < ACF < 10
- ACF > 10.

Colonoscope CF-Q-165 L (not HD),“biopsy forecps” FB-240 U Olympus and catheter type spray Olympus by Olympus company were used in the examination. All 131 subjects underwent a full colonoscopy.

After routine colonoscopy, the rectal mucosa was stained with 0.25% solution of methylene blue from the serratus line to the medial Houston’s valve. ACF were assessed using the endoscopic criteria established by Roncucci [28]. In the statistical analysis, numerical data were presented and real numbers, range of arrhythmic means, mean standard deviation and results of probability distribution. The Student’s t test and the U test were applied in order to determine the significance of differences of means and frequency of events in both groups. Statistica 7.1 and Excel 2010 were used.

## Results

The study group – 73 women and 58 men.

The mean age in the study group was 67 years for women and 52 years for men.

**Fig. 1:**
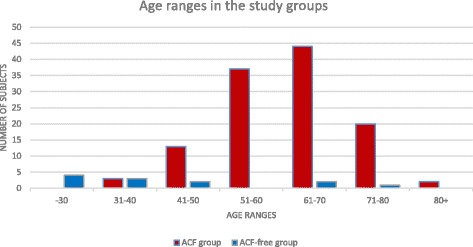
Age ranges in the study groups

The age distribution in the groups was found “normal”, which enabled the evaluation of ACF incidence and characteristics according to age. The incidence of ACF in the study population correlates with the incidence of CRC in the entire population (Fig. 1).

**Fig. 2:**
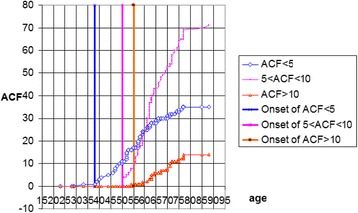
Quantification of ACF in various age groups

The data presented in Fig. 2 demonstrate that single ACF occur in the youngest individuals (ACF < 5). Since the age of 38 years, the number of ACF gradually increases to show a decreasing tendency since the age of 60 years.

The number of 5 < ACF < 10 occurs slightly later, since the age of 50 years, and dynamically increases reaching the maximum at the age of 62 years, subsequently the increase is proportional. ACF > 10 occur at a more advanced age (55 years) and their number gradually increases with age (linear increase). The maximum number is observed at the age of 77 years.

**Fig. 3:**
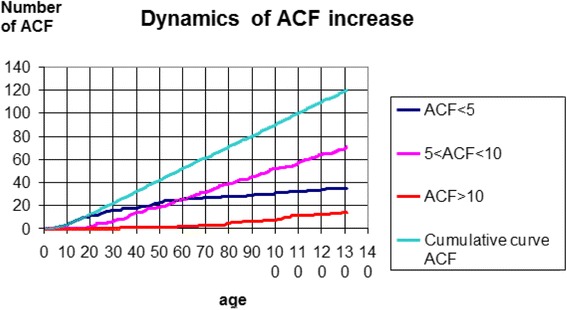
Dynamics of ACF increases in individual age groups

Figure 3 shows that the number of 5 < ACF < 10 increases most dynamically. The cumulative curve (green) reveals a gradual increase in the number of ACF dependent on age. During the first period, the rate of increase was dynamic (0–50); in the second period, the rate of increase was constant. The range of 5 < ACF < 10 predominated in the group aged 51–60 years and the group aged 61–70 years.

**Fig. 4:**
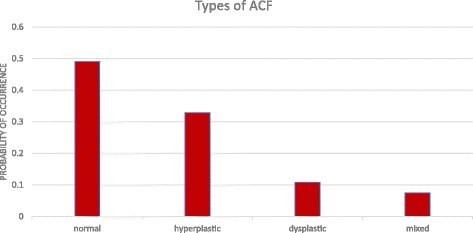
Types of ACF in the study group

In the examined group the most common finding was ACF normal and the rarest one was mixed (Fig. 4).

**Fig. 5:**
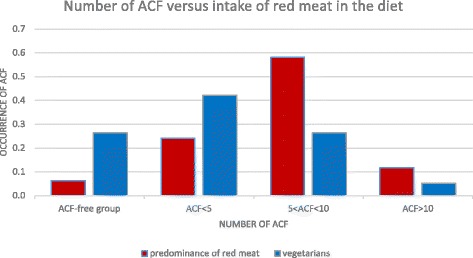
Quantificiation of ACF according to diet

In patients in whose diet red meat is prevalent or it comprises mostly of red meat there were more cases of ACF. In patients who consume a lot of vegetables, fruit, poultry and fish the number of ACF cases was lower (Fig. 5).

**Fig. 6:**
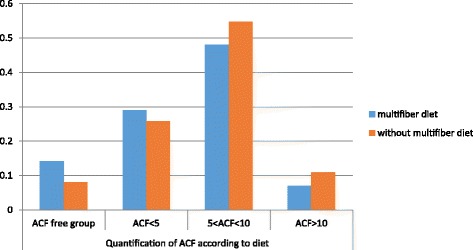
Quantificiation of ACF according to diet(continued)

In the research group where the people used a high-fiber diet there is a lower number of ACF cases while in the group who did not follow such diet the number rises which is especially visible when the number of ACF cases is higher than 5 (Fig. 6).

The probability of higher numbers of ACF (5 < ACF < 10 and ACF > 10) was observed in patients with colon diverticula.

**Fig. 7:**
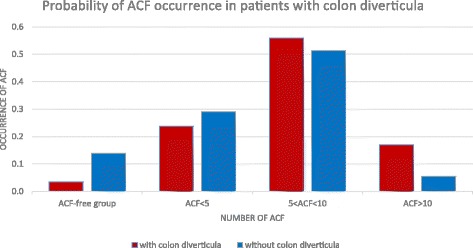
Quantification of ACF in patients with colon diverticula

In 72 people in the examined group diverticula were found in the large intestine (Fig. 7).

**Fig. 8:**
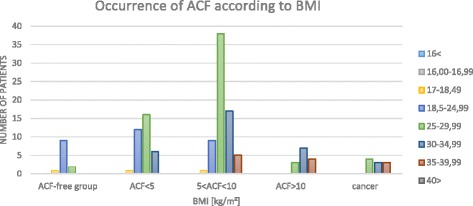
Occurrence of ACF according to BMI

In patients with higher BMI, the number of ACF is higher (Fig. 8).

## Discussion

The development of colorectal cancer and its precursors is a complex and multi-stage process. Neoplasia progresses over time. ACF are the most common, endoscopy-identifiable colonic epithelial neoplasms [20]. In our study, ACF occurred sporadically since the age of about 40 years; predominantly single foci were found [20]. Since the age of 45 years, the number of ACF rapidly increased to reach the plateau phase about the age of 60 years and subsequently slowly decreased. Similar incidences of ACF according to age have been reported by other authors. [21–26].

Takayama et al. [21] have found the highest incidence of 5 or less colorectal ACF in individuals aged 40–50 years, which was also confirmed in out study.

Not much is known about the factors that initiate and stimulate the growth of ACF in humans. It seems highly likely that since colorectal cancers develop in the ACF-adenoma-cancer sequence, the same factors that induce the development of cancer should cause the development of ACF. With age, an individual acquires new mutations. Statistically, the number of ACF and malignant tumours increases in older people. The studies evaluating the epidemiology of ACF have demonstrated that similar factors promote the development of ACF and colorectal cancer [21–23, 26–32]. It is essential that the number of ACF does not increase in patients over the age of 80 years. On the contrary, their number decreases. It is more than likely that the mucous membrane in the distal fragment of the colon atrophies in individuals > 80 years of age and regeneration is not so intense as in younger people. The fact that colorectal cancer is frequently diagnosed in this age group is rather associated with a slow growth of cancer at advanced age, which has not been diagnosed earlier.

Why is the gradual increase in the number of ACF observed about 40–50 years of age and a decreasing tendency found after 60–65 years of age? The probability of cell kinetics and risk factors for the development of ACF and CRC supports the hypothesis that ACF are pre-cancerous changes. According to Figueoredo et al. [33], apoptosis is markedly reduced in ACF and patients with concomitant CRC aged more than 50 years. Roncucci et al. [26] states that one type of cell kinetics disorder in ACF in people suffering from CRC which is the rise of total cell proliferation in ACF. In other authors’ research the focus is on the fact that the cell division line moves up the crypt, towards its surface. In older people, ACF become larger and are markedly bigger compared to those in younger individuals [21–23, 26]. Larger lesions contain higher numbers of pathological crypts in the focus, thus the number of cells with proliferation abnormalities is higher. The repair of larger lesions by efficient mechanisms of elimination of “damaged” cells can be more difficult.

Do lifestyle and diets affect the incidence of colorectal ACF? Analysis of the number of ACF in individuals with bad dietary habits, those overweight and obese reveals higher numbers of ACF in these patients, as compared to the “proper” diet group.

According to Bruce et al. [34], the type of diet and the amount of calories affect significantly the occurrence of ACF as well as benign and malignant neoplastic lesions in the colon. Numerous studies have confirmed a clear correlation between the occurrences of ACF, increased BMI, excessive subcutaneous and visceral fatty tissue and excessive amounts of triglycerides circulating in blood. An increase in the number of ACF is particularly distinct in individuals with excessive amounts of visceral fat; the visceral adipose tissue is considered “an endocrine organ” secreting adipocytokines, such as TNF-α, leptin and adiponectin that can play an important role in the process of carcinogenesis. Leptin, a satiety hormone, informs the central nervous system about energy requirements mediated by neuropeptide Y (NPY) produced in the subthalamic nucleus. By triggering the feeling of satiety, NPY reduces the intake of food products and simultaneously increases the consumption of energy. Impaired production of this hormone or insensitivity of receptors for this hormone often leads to overweight and obesity. Under normal circumstances, leptin also reduces the secretion of insulin, which in some cases is a factor indirectly stimulating the colonocytes. Individuals with bad dietary habits frequently consume meals in the evening or at night, which can be associated with impaired secretion of leptin. During these hours, the highest level of leptin is observed, thus the appetite should be reduced. The experimental study has demonstrated that leptin can be a growth factor for colonocytes [35–38].

Proper nutrition and physical activity are frequently mentioned as protective factors of carcinogenesis. Impaired energy metabolism develops due to imbalance in the ratio between energy supply and consumption. The endocrine system is highly responsible for maintenance of this balance. Excessive amounts of circulating insulin in serum and an excess of energy available for colonic epithelial cells stimulate the pathway of signals for excessive proliferation, particularly favouring the cells with defects in cell cycle control system, both the pathway of chromosomal and microsatellite instability. Giovancucci et al. [39] and McKeown [8] have suggested that hyperinsulinaemia with hypertriglyceridaemia enhances the promotion of growth and divisions of neoplastic cells.

Excessive amounts of circulating glucose and fatty acids increase the tissue breakdown, which can increase the production of free radicals that in turn can increase the number of spontaneous mutations in colonocytes.

The other factors responsible for initiating of the neoplastic process are iron compounds, which are derived from dietary red meat and accumulate excessively in the colon. The faeces with a high content of iron form complexes with biliary pigments (bilirubin, biliverdin) and are capable of inducing the adverse Fenton’s phenomenon, which results in the formation of superoxide radicals. The presence of superoxide anion-radicals causes the formation of peroxide hydrogen and subsequently of a hydroxyl radical, which has serious biochemical implications. A hydroxyl radical is so reactive that it is formed and reacts in the same place. Peroxide hydrogen, on the other hand, is a stable molecule and can migrate through the cell membranes, which has the potential of damaging DNA and proteins of colonocyte organelles [18, 19].

The surface of colonocytes forming ACF and adenomas is immature and deficient in microvilli. ACF are deficient in protective mucus whose quality is changed, which additionally favours the damage to epithelial cells and exposes them to other mutagenic factors.

The above effects are particularly visible in villous and tubulo-villous adenomas in which potassium ions escape to the crypt lumen, the quality of mucus is impaired and the mucus barrier is distinctly weakened. The mucus barrier defect exposes deeper layers of the colonic wall, thus exposing them to the action of bile acids, fatty acids and toxic bacterial products that additionally damage the intestinal barrier and the epithelium [34]. Vaccina et al. using scanning electron microscopy showed that the mucosal surface among aberrant crypts was flattened because of loss of microvilli [40]. High-fat and high-protein diets change the faecal pH into alkaline, which enhances toxic and carcinogenic effects of various chemical compounds present in the faeces. Therefore, high dietary plant fibre intake is essential, thanks to which fatty acids are produced in the colon, which reduce faecal pH. Dietary fibre can reduce the risk of colorectal cancer. High-plant fibre intake shortens the time of intestinal passage, which in turn shortens the chyme-colonic surface area contact. The dietary carcinogenic compounds and those formed during digestive processes have less time to exert adverse effects on colonocytes. Another beneficial effect of dietary fibre on the colon is an increase in stool volume and binding of cholesterol and bile acids, which prevents their conversion into other carcinogenic compounds [41, 42].

Moreover, diets rich in vegetables and fruit provide antioxidants, which limit the adverse effects of local inflammatory responses. Due to the lack of dietary vegetables and fruit, the stimulation of local inflammatory response results in increased levels of COX-2. Its levels in the monocytes, macrophages, epithelial cells and fibroblasts increase multiple times. COX-2 catalyses the synthesis of prostaglandins, prostacyclins and thromboxanes from arachidonic acid, that are the substances stimulating the proliferation and inhibiting apoptosis of abnormal cells [43].

The above hypothesis has been confirmed by studies regarding ACF, adenomas and colorectal cancers exposed to UV radiation emitting the spectrum characteristic of lipofuscin and ceroid, which suggests that these cells are under the oxidative stress [34]. As a result, the number of factors stimulating proliferation increases and genetic instability of tissues is enhanced. The above-mentioned processes take place focally and initiate the mutation in one or several cells. If the processes are not inhibited, the cell clone expands in ACF and the carcinogenesis cascade is activated. If the processes are inhibited and genetic errors repaired, ACF should not progress to adenomas.

Besides obesity and bad dietary habits, physical activity is a relevant protective factor of colorectal cancer. In about 12–14% of cases, low physical activity is considered to affect significantly the development of colorectal cancer. The literature data show that 4–5-h vigorous physical activity a week is optimal for the body. It is favourable for maintenance of proper body weight and good motor function of the intestines; moreover, it reduces the levels of circulating insulin and IGF and maintains proper values of prostaglandins, which also substantially affect the formation of ACF [44]. Diets and lifestyle significantly influence the development of ACF, benign and malignant tumours of the colon, which has been confirmed by some authors [2, 3, 7, 35, 45, 46].

Another important issue is the development of ACF and colorectal cancer in patients with colonic diverticula. The diverticula are non-neoplastic lesions and do not increase the risk of colorectal cancer. However, the changes in the muscular layer of the colon result in shortening and thickening of the circular muscle bands, their contraction and division of the colon into segments, hence the intestinal passage becomes shorter and water absorption increases. The above changes favour constipations; as a result, the contact of faeces and potential carcinogens in them with the intestinal mucosa is prolonged, which can cause more mutations and increase the frequency of abnormal cell divisions in colonocytes.

Shpitz et al. [22] and Dolara et al. [47] have demonstrated increased incidences of ACF in colonic diverticula compared to normal colonic walls. According to Roncucci et al. [28], about 60% of patients with diverticula have ACF. Yokota et al. [48] have reported that patients with colonic diverticula predominantly have 1–10 ACF. Their data do not define the type of ACF that most commonly accompanies diverticula. In our study, higher numbers of ACF (5 < ACF < 10 and ACF > 10) are observed in patients with colonic diverticula, which is confirmed by other authors (Table 1).

**Table 1:** Occurrence of ACF according to BMI

BMI [kg/m^2^]	ACF-free group	ACF < 5	5 < ACF < 10	ACF > 10	cancer
16<	0	0	0	0	0
16–16.99	0	0	0	0	0
17–18.49	1	1	1	0	0
18.5–24.99	9	12	9	0	0
25–29.99	2	16	38	3	4
30–34.99	0	6	17	7	3
35–39.99	0	0	5	4	3
40>	0	0	0	0	0

## Conclusions

Age significantly affects the number of colorectal ACF. Single ACF are found in patients below the age of 40 years; the highest numbers are observed in 50–70-year-old patients. Above the age of 70 years, the number of ACF does not increase. Diets can markedly increase the risk of colorectal ACF, which is particularly visible in patients whose regular diets are not fibre-high, in obese individuals and those with colonic diverticula.
